# Perspectives on the Use of Biopolymeric Matrices as Carriers for Plant-Growth Promoting Bacteria in Agricultural Systems

**DOI:** 10.3390/microorganisms11020467

**Published:** 2023-02-13

**Authors:** Jéssica F. Pereira, André Luiz M. Oliveira, Daniele Sartori, Fabio Yamashita, Suzana Mali

**Affiliations:** 1 Department of Biochemistry and Biotechnology, State University of Londrina—UEL, Londrina 86057-970, PR, Brazil; 2 Department of Food Science and Technology, State University of Londrina—UEL, Londrina 86057-970, PR, Brazil

**Keywords:** biofertilizer, biopesticide, biopolymer, sustainable agriculture

## Abstract

The subject of this review is to discuss some aspects related to the use of biopolymeric matrices as carriers for plant-growth promoting bacteria (PGPB) in agricultural systems as a possible technological solution for the establishment of agricultural production practices that result in fewer adverse impacts on the environment, reporting some promising and interesting results on the topic. Results from the encapsulation of different PGPB on alginate, starch, chitosan, and gelatin matrices are discussed, systematizing some advances made in this area of knowledge in recent years. Encapsulation of these bacteria has been shown to be an effective method for protecting them from unsuitable environments, and these new products that can act as biofertilizers and biopesticides play an important role in the establishment of a sustainable and modern agriculture. These new products are technological solutions for replacing deleterious chemical fertilizers and pesticides, maintaining soil fertility and stability, and improving crop productivity and food security. Finally, in the near future, scale-up studies will have to provide new information about the large-scale production of these materials as well as their application in the field under different biotic and abiotic stress conditions.

## 1. Introduction

The current scenario with regards to the continuous increase in world population growth has created concerns related to food production and the environmental impact of agricultural practices. Around 2.3 billion people in the world were moderately or severely food insecure in 2021 as a result of the COVID-19 pandemic in 2020–2021, while the ongoing war in Ukraine represents an additional challenge for ending hunger at a global level. The Russian Federation and Ukraine are among the most important producers of agricultural commodities in the world; these two countries together supplied 30 percent and 20 percent of global wheat and maize exports, respectively [1].

This scenario becomes more challenging when taking into account the climate changes experienced in the twenty-first century and the important global changes that have been encountered during the past decades. According to Abbass et al. [2], “climate change is characterized based on the comprehensive long-haul temperature and precipitation trends and other components such as pressure and humidity level in the surrounding environment. Besides, the irregular weather patterns, retreating of global ice sheets, and the corresponding elevated sea level rise are among the most renowned international and domestic effects of climate change.”

Climate changes at a global level result in several environmental biotic and abiotic stresses, which are threatening agricultural crop production [3,4]. According to the report published by the Intergovernmental Panel on Climate Change (IPCC) [5], climate change impacts are negatively affecting agriculture and crop production on a global scale, slowing the productivity growth of agriculture over the past 50 years. Global warming has been affecting soil health, increasing crop vulnerability to pests and diseases, adding pressure to food production systems, and undermining food security. Additionally, the availability and quality of water for population and agricultural uses should decrease as a consequence of the increased frequency of extreme events, such as floods, droughts, and heat stresses, resulting in adverse impacts on food availability and prices, as well as exposing populations to water scarcity.

Sohail et al. [6] report that agriculture faces a serious threat from climate change and that there is a growing need to develop new strategies to protect future agricultural production. In this sense, the scientific community and civil society have been directed to propose political and innovative solutions to mitigate these effects. The development of agricultural production systems based on sustainability can be one of the viable alternatives to this global challenge.

Sustainable agriculture includes several approaches and practices that are associated with food security, allow for more efficient use of natural resources, and contribute to accomplish some of the Sustainable Development Goals (SDGs), especially those associated with poverty, hunger, responsible consumption and production, climate change, and ecosystems [7,8]. Within this context, the establishment of a sustainable agriculture production system must attend to the needs for improved crop productivity with the maintenance of soil fertility and ecosystem services to reach food security for the current and future generations. The use of plant-growth promoting bacteria (PGPB) as agricultural inputs can meet the requirements for sustainability as an eco-friendly alternative to the use of potentially deleterious chemical fertilizers, herbicides, fungicides, and insecticides, while improving plant productivity and health [4,9,10,11,12,13,14].

In recent decades, agricultural biotechnology research has been directed towards the development of microbial inoculants containing one or more PGPB strains [12,13]. The use of several biopolymeric matrices as carriers for PGPB has been reported as a promising strategy to obtain new inoculant formulations, protecting microbial cells and their metabolites from adverse external conditions and ensuring the uniform and gradual release of cells near the targeted site [15,16,17,18,19,20,21,22,23].

The subject of this review is to discuss some aspects related to the use of biopolymeric matrices as carriers for PGPB in agricultural systems as a possible technological solution for the establishment of an agriculture that results in less adverse impacts on the environment, reporting some promising and interesting results on the topic. Results from the encapsulation of different PGPB on alginate, starch, chitosan, and gelatin matrices are discussed, systematizing some advances made in this area of knowledge in recent years.

## 2. Plant Growth-Promoting Bacteria (PGPB) and Their Mechanism of Action

Microbial populations are found in diverse ecological niches, while soil microbial communities play a pivotal role in this ecosystem’s functioning and services. Soil microbial diversity is influenced by a myriad of variables, including plant cover. The large variety of compounds released into the soil through plant roots provide nutrients for the microorganisms living in the root vicinity, creating a distinct site from the bulk soil referred to as the rhizosphere, which comprises the volume of soil surrounding plant roots [13,24,25,26,27,28,29,30]. PGPB are nonpathogenic microorganisms that are natural soil inhabitants and are enriched in the rhizosphere. They can colonize plant internal compartments and surfaces, establishing mutualistic relationships with the host plant that result in several beneficial effects for both partners [24,25,26,27,28,29,30].

PGPB have been used as biofertilizers and biopesticides for decades, improving plant health and promoting plant growth while protecting them from biotic (plant pathogens, including fungi, bacteria, viruses, insects, and nematodes) and abiotic (drought, salinity, flooding, extreme temperatures, pH, light intensity, and the toxicity of accumulated heavy metals) stresses by several different mechanisms, modulating the plant metabolism at physiological, morphological, biochemical, and molecular levels. The most studied abiotic stress conditions are drought and salinity [3,4,10,11,13,24,25,26,27]. PGPB can positively affect plant development through direct or indirect mechanisms [13,24,25,26,28,29].

Direct mechanisms are those that result in the direct promotion of plant growth, such as biological nitrogen fixation, phosphate solubilization, and the synthesis of phytohormones, including auxins (indole-3-acetic acid (IAA)), gibberellins, zeatin, and abscisic acid, which result in enhanced nutrient uptake and plant development. Additionally, some PGPB can modulate the concentration of phytohormone ethylene by synthesizing 1-aminocyclopropane-1-carboxylate (ACC) deaminase, an enzyme that cleaves the compound ACC, which is the immediate precursor of ethylene in all higher plants. Bacterial ACC deaminase helps plants reduce many of the symptoms related to stress exposure by reducing endogenous ethylene synthesis. In general, phytohormones produced by PGPB play an interdependent role in the integration of signaling pathways involved in the modulation of plant metabolism to a given environmental condition [3,4,13,24,25,26,29].

Indirect mechanisms include the production of siderophores, antibiotics, enzymes that can break down fungal and insect larval cell architecture (glucanases and chitinases), and the synthesis of small volatile organic compounds that are toxic to many phytopathogens. In general, these indirect mechanisms are responsible for protecting plants from biotic stress [3,4,13,24,25,26,29].

In addition, PGPB can improve plant tolerance against water stress via different pathways, such as reprogramming the root architecture, which results in increased root surface, favoring nutrient and water acquisition [24,27]. PGPB can also induce the accumulation of osmoprotectants inside plant cells, like trehalose, proline, glycine, and phenols, among others, as well as increase the production of exopolysaccharides at the root surface, which can provide protection against desiccation during drought stress by enhancing water retention [3,4,13,24,25,26,29,30].

**Figure 1:**
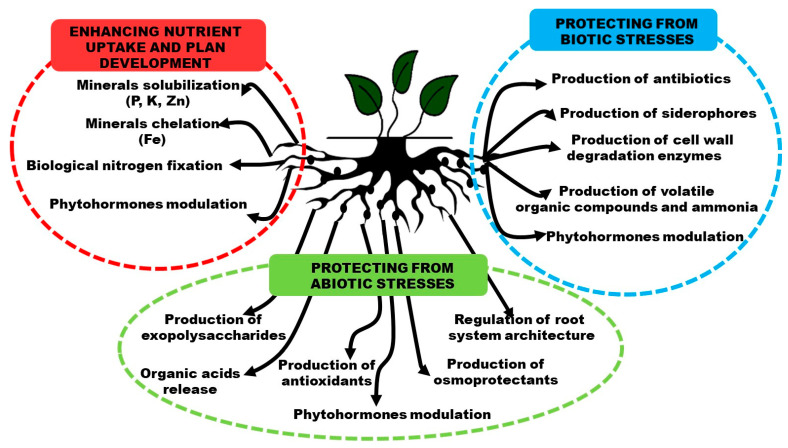
Overview of the major mechanisms used by PGPB to enhance nutrients uptake and plant development, as well as to protect plants from biotic and abiotic stresses.

Figure 1 presents an overview of the major mechanisms used by PGPB to enhance nutrient uptake and plant development, as well as mechanisms related to protection from biotic and abiotic stresses. It is important to highlight that these mechanisms are associated and interconnected [3,29,30]. Stegelmeier et al. [29] reported that a specific strain can act via multiple mechanisms. The active mechanism(s) expressed in a given plant-PGPB interaction can be a consequence of plant stage and growth conditions, including soil composition, temperature, and the presence or absence of stressful compounds and/or phytopathogens in the soil.

Several reviews have been published in the last few years, mostly concentrating on the mechanisms of action of PGPB and how these microorganisms act under different biotic and abiotic stress conditions [3,4,10,11,13,14,15,23,24,25,26,27,28,29,30,31,32,33,34,35,36,37,38]. *Acinetobacter*, *Agrobacterium*, *Arthobacter*, *Azotobacter*, *Azospirillum*, *Bacillus*, *Bradyrhizobium, Burkholderia*, *Enterobacter*, *Erwinia*, *Frankia*, *Klebsiella*, *Micrococcus*, *Paenibacillus*, *Pseudomonas*, *Rhizobium*, *Serratia*, and *Thiobacillus* are among the PGPB genera reported in the literature [13,15,23,24,26,36,37,38].

Table 1 lists some examples of the main mechanisms of action attributed to PGPB and the respective microorganisms described in the literature associated with these mechanisms. It can be seen that PGPB act through multiple mechanisms in general, making this type of microorganism an effective and promising alternative in the search for candidate strains to be used as inoculants towards a sustainable agriculture with less environmental impact.

While several PGPB are extensively cited in literature, a small number of strains have been commercialized as bioinputs for agricultural practice, possibly because of their inappropriate formulation [3,23,36]. However, the number of commercial strains continues to increase, with novel ones emerging as potential inoculants [3,7,23,24,25,26]. Cao et al. [4] reported some recent examples of commercial applications of PGPB, including *A. brasilense*, *B. fimus*, *B. megaterium*, *B. subtilis*, *P. fluorescens*, and various *Rhizobium* sp.

The *Azospirillum* genus contains the best characterized PGPB, being able to fix biological nitrogen and produce phytohormones, including auxins, such as IAA, and gibberellins [15]. According to Santos et al. [37], inoculants containing *A. brasilense* have been commercialized for more than 20 years: since 1996 in Argentina, 2002 in Mexico, and 2009 in Brazil. In 2020, the number of commercialized doses of inoculants containing this PGPB reached 10.5 million [61]. Some countries have created laws to regulate the commercialization of these products, ensuring their safety and quality [37]. In Australia, since 2010, rhizobial inoculants that have been tested by the Australian Inoculants Research Group (AIRG), which displays a registered trademark called the “Green Tick Logo,” indicating that an inoculant has been independently tested and meets Australian quality standards [37,62].

Regulatory standards in Brazil were established in 2004 based on the Australian standard and updated in 2011 [37,63,64]. In Brazil, a list of PGPB strains was authorized for inoculation, and bacterial species must present 1 × 10^9^ CFU (colony-forming units) per gram or mL until the expiration date, which must be at least 6 months [64,65].

According to the European Union (EU) Regulation, only *Rhizobium*, *Azospirillum*, and *Azotobacter* genera have been approved to be used as biostimulants on the basis of Regulation (EU) 2019/1009 [66,67]. According to the EU Regulation, a fertilizing product can contain “microorganisms, including dead or empty-cell microorganisms and non-harmful residual elements of the media on which they were produced, which have undergone no other processing than drying or freeze drying.”

The development of products on an industrial scale still has some technological challenges to be overcome, including the viability of microorganisms during their application so that they can exert beneficial effects during plant growth. Bashan et al. [36] defined some important terms within this theme: “(1) Carrier refers to the abiotic substrate (solid, liquid, or gel) that is used in the formulation process; (2) Formulation refers to the laboratory or industrial process of unifying the carrier with the bacterial strain; and (3) Inoculant refers to the final product of formulation containing a carrier and a bacterial agent or consortium of microorganisms.” These authors stressed that the use of PGPB without a suitable carrier can result in a decrease in bacterial population, with negative impacts on the rhizosphere PGPB population. 

The development of high-quality inoculant formulations is a huge challenge for the consolidation of crop inoculation technology. The use of a biopolymeric matrix as a carrier for PGPB can bring some advantages related to the increase of bacterial survival in soil near the target plant, increasing the inoculation efficiency in comparison with other techniques, including seed inoculation or direct soil spreading of bacterial suspensions [14,16,17,68]. The use of biopolymeric matrices as carriers of PGPB can be considered a technological solution for this sector; however, the development of these novel formulations needs to consider some aspects, including cost, availability of the raw materials, the possibility of large-scale production, and the efficiency of encapsulated microorganisms.

## 3. Biopolymeric Matrices as Carriers for PGPB

Biopolymers are polymers from renewable sources, and a large portion of them are biodegradable, including alginate, starch, gelatin, and chitosan, among others [69]. Biopolymers have been extensively studied as potential raw materials for replacing synthetic polymers from non-renewable sources, in different industrial sectors including their use in agriculture as carriers of PGPB [68,69].

The immobilization and encapsulation of PGPB cells consists of entrapping them in a polymeric material, which results in the protection and stabilization of cell structure, potentially enhancing their viability and stability in the production, storage, and handling of cultures and conferring additional protection during rehydration [68,69,70]. In the development of an inoculant formulation, the carrier corresponds to the major fraction, and according to Bashan et al. [36], there are five types of carriers: (I) Soils; (II) Lignocellulosic residues from industrial and agricultural origins; (III) Inert materials, including biopolymers; (IV) Plain lyophilized microbial cultures; and (V) Liquid inoculants.

Carrier materials are chemically stable materials that provide a protective niche. They must be nontoxic, readily available, low-cost, preservative-free, able to maintain humidity, and stable when stored at room temperature for long periods [14,16,68,70]. Bashan et al. [36] also stressed that an ideal carrier must be easy to handle, ensuring controlled release of the microorganism, and it has to be easy to manufacture and to combine with other additives or nutrients.

Several techniques have been described in the literature for the encapsulation of microbial cells in a biopolymeric matrix, prolonging the shelf life of a usable strain under biotic and abiotic stress conditions. Ionic gelation, emulsification, and spray drying are the most important techniques for the encapsulation of PGPB [16]. In addition to the carrier polymers, additives can reduce the cost, improve stability, survival, encapsulation efficiency, mechanical properties, and swelling properties of these materials. Some fillers, such as polymers (starch, gelatin, and chitosan, among others), clays (bentonite, perlite, and kaolin), osmoprotectants (sugars, such as trehalose, sucrose, glucose, and fructose), and nutritional compounds can also be added [16,36,71,72].

### 3.1. Polymeric Matrices Based on Alginate

Alginate is an anionic polymer that is present on the cell walls of brown algae. Some bacteria can also produce alginate, but commercial alginate is extracted from algae biomass [73,74,75]. Alginate has an unbranched chain consisting of 1,4-β-D-mannuronic and 1,4 α-L-guluronic acids, whose carboxylate groups carry a net negative charge. The ratio of mannuronic and guluronic acid can vary in composition and sequence depending on the source [16,73].

The main characteristic of alginate is gelation in contact with calcium ions (Ca^2+^), resulting in hydrogels with an ionic crosslinked three-dimensional matrix, which generally are obtained as beads, capsules, micro- and nanocapsules [14,16,76]. Alginates are biodegradable, and the presence of reactive carboxylic groups and their status as GRAS (Generally Recognized as Safe) by the FDA (Food and Drug Administration-USA) make them ideal candidates to be used as carriers for PGPB [73,74]. Alginates are extensively studied as PGPB carriers [76,77,78,79,80,81,82,83,84,85,86,87,88,89,90,91,92,93,94,95,96,97,98,99] to be used as biofertilizers or biopesticides. Most articles report encapsulation of *A. brasilense* [22,77,78,80,82,85,96,98] as a biofertilizer, but other microorganisms, such as *A. lipoferum* [86], *P. fluorescens* [78,79,81], *Pseudomonas* sp [87,92], *P. putida* [90,95], *P. libanensis* [93], *P. corrugata* [81], *Serratia marcescens* [87], *Klebsiella oxytoca* [84], *Rhizobium* ssp. [89], *B. subtilis* [21,90,91] and *B. pumilus* [94] are also studied.

Jung et al. [99] and Fages [100] were the first authors to report the use of alginates as carriers for *Rhizobium* and *Azospirillum*, respectively, and since then, many of the beneficial effects of this biopolymer as a carrier for PGPB have been confirmed. Bashan et al. [78] reported that *A. brasilense* and *P. fluorescens* encapsulated in alginate beads can survive over long periods, pointing out that the porous alginate matrix protects cells against mechanical stress and also that bacteria tend to occupy the pores present in the polymeric matrix. Schoebitz et al. [85] reported that dried alginate beads containing *A. brasilense* presented 76% of viable cells after one year of storage. Zago et al. [96] reported that the growth of *A. brasilense* was maintained for 90 days when this microorganism was encapsulated in alginate beads, ensuring better cell viability. Additionally, Bashan et al. [22] reported that a minor disadvantage can affect the encapsulation of *A. brasilense* in alginate beads; the crosslinking of the alginate-calcium complex with the bacterial cell wall can result in death of a large number of the bacteria, however, it is easy to avoid by a secondary incubation of alginate beads in a fresh growth medium. The surviving bacteria will multiply and restore the concentration to that in the original growth medium.

*Pseudomonas* sp. has been entrapped in the alginate beads along with the salicylic acid and zinc oxide nanoparticles by Panichikkal et al. [92], and they reported superior plant growth-promoting and biocontrol properties of the encapsulated *Pseudomonas* sp. on rice seedlings by comparing them with the free-living microorganisms. Souza-Alonso et al. [93] studied the encapsulation of *P. libanensis* using polymeric beads of alginate and observed that cell viability was maintained for up to 90 days and that the alginate beads were progressively dissolved in the plant rhizosphere.

Hernández-Montiel et al. [95] reported that the use of PGPB as biofertilizers could be an alternative in agricultural management and sustainable production of tomatoes, and they stressed that the immobilization of *P. putida* in alginate microcapsules conferred protection and gradual release, improving adhesion, permanency, and colonization of cells on the roots, promoting a better effect on the productivity of tomato plants.

Two *P. fluorescens* strains were encapsulated in alginate-gelatin beads with different concentrations of gelatin, and they were employed in the inoculation of potato plants as biopesticides, resulting in reduced fungal growth and incidence of diseases, protecting plants from harsh soil conditions, and favoring the PGPB establishment in the rhizosphere. The mechanisms associated with the beneficial effects of microorganisms encapsulated in alginate beads include the production of antifungal agents, such as antibiotics and enzymes, the production of siderophores, phosphate solubilization, and ACC deaminase activity [98].

Saberi-Riseh and Moradi-Pour [91] reported that encapsulation significantly improved the survival rate of *B. subtilis* compared to the free form of the microorganism. Young et al. [21] reported the encapsulation of *B. subtilis* in alginate beads enriched with humic acid, and they stressed that the encapsulated bacteria had high viability upon storage for 5 months, promoting successfully the plant growth of lettuce. *B. megaterium* was encapsulated in alginate microcapsules to be used as a biopesticide against *Rhizoctonia solani*, which is an important phytopathogen in rice plants. Encapsulated alginate cells had increased resistance to UV radiation, which was validated when the product maintained its efficacy to inhibit the mycelial growth of *R. solani* after 48 h of treatment with UV radiation [97].

In general, polymeric matrices based on alginate are prepared in an aqueous medium employing concentrations between 0.5 and 3.0%, and the most commonly used crosslinking agent is calcium chloride (CaCl_2_) in concentrations ranging from 1.0 to 2.0%. Additional components can improve the mechanical and swelling properties of alginate materials, resulting in higher efficiency of encapsulation [100,101], such as fillers and osmoprotectants.

Starch is a low-cost biopolymer, and its addition as a filler to alginate formulations has been reported as a successful strategy for improving the beads’ resistance to physical stress and protecting against UV radiation [20,85,102]. The survival of *A. brasilense* during the encapsulation process can be improved by incorporating starch as filler in the alginate beads’ composition, especially for microorganisms that can use starch as a carbon source [85]. The use of trehalose as an osmoprotectant additive also improved cell viability during storage of *A. brasilense* encapsulated in alginate beads [96].

Rohman et al. [102] reported that alginate beads can be considered a promising carrier for biofertilizer release, but some drawbacks are still observed, including low mechanical strength, poor appearance, high porosity, and consequently an inadequate cell protection. Blending alginate with starch helps beads retain their spherical shape after drying and improve the entrapment of bacterial cells in the polymeric matrix, reducing cell loss.

### 3.2. Polymeric Matrices Based on Starch

Starches from different sources have been the object of intensive academic and industrial study for several reasons, including their renewable source, biodegradability, low cost, and wide availability [103,104,105]. Starch presents important characteristics as a potential biopolymeric carrier for PGPB, which include high solubility, controlled release, and nontoxicity [103]. Starch also presents well-known properties for film formation and for the ability to obtain micro- and nanoparticulate systems and gels [105], making this biopolymer a suitable material to be used as a carrier for bioactive compounds and microorganisms.

Starch can be obtained from cereals, roots, tubers, legumes, and immature fruits [106]. Starch is a homopolymer of α-D-glucose units consisting of a mixture of two fractions, amylose and amylopectin, with α-(1→4) linkages in the linear amylose and α-(1→4) linkages and ∼5–6% of α-(1→6) branch linkages in amylopectin [107]. The ratio between amylose and amylopectin depends on the starch source; variations in the proportions between these fractions can result in starch granules with very different physicochemical and functional properties, which can affect their industrial applications.

The application of starch to obtain biodegradable polymeric matrices is based on the chemical, physical, and functional properties of amylose and amylopectin to form gels and their ability to form a continuous polymeric network maintained by hydrogen bonds. These matrices can be obtained in aqueous solutions, with starch concentrations ranging from 1.5 to 3.0% [18,108,109,110] or by other processes, such as thermoplastic extrusion [17,108]. Extrusion is a high-temperature, short-duration process with the advantages of high versatility and the absence of effluents; additionally, it is easy to adapt to large industrial scales [17].

The use of starch as a PGPB carrier is still little reported in the literature, although in recent decades its use for the encapsulation of bioactive compounds has been extensively discussed and studied [105], such as particles to encapsulate curcumin for pharmaceutical applications [111,112], as films to encapsulate antioxidants for food packaging [113,114], and as films to enhance the stability of phenolic compounds to be used in food technology [115,116].

While the use of pure starch is not reported for obtaining polymeric matrices as carriers of PGPB, starch is extensively reported as a filler in alginate matrices for PGPB encapsulation, providing protection to their cells and allowing diffusion of some important compounds, such as micronutrients and metabolites, in inoculant formulations. Considering their hydrophilic character, starch molecules can retain immense amounts of water in the interstitial sites of their polymeric network, contributing to bacterial survival and the effectiveness of inoculation strategies [14,85,96,102,103,104,105].

The main disadvantage of obtaining stable starch matrices for the encapsulation of microorganisms can be considered their poor mechanical performance [103,105,107,108,109], and in order to improve these characteristics, the incorporation of plasticizers, fillers, and the mixture of starch with other biodegradable polymers can be considered feasible strategies for the development of polymeric matrices for PGPB encapsulation with commercial potential. Mali et al. [110] reported that plasticizers can reduce intermolecular forces and increase the mobility between polymer chains, reducing possible discontinuities and brittle zones and thus resulting in materials with improved mechanical properties. Glycerol and sorbitol are the most commonly employed plasticizers in combination with starch.

Some authors reported the use of starch as a major component in mixtures with other polymers and materials to obtain effective PGPB carriers [17,18,22]. Marcelino et al. [17] studied the production of solid formulations obtained by extrusion for PGPB inoculation containing starch as major component (58.5–72.4%), with addition of sugarcane bagasse (9.4–23.0%), and other minor components (glycerol, rock phosphate, crystal sugar, powdered skim milk and yeast extract). The solid polymeric matrices could maintain the viability of *A. brasilense* cells for up to 120 days at room temperature. The solid formulations were applied to the soil 10 days before the maize was sown, contributing to the plant’s growth by providing a beneficial environment that supports the viability of bacteria in soil for longer periods than those observed for liquid inoculant formulations. Additionally, minor nutritional elements and/or bioactive compounds could be added to these solid formulations, favoring plant performance and productivity in the field.

Vercelheze et al. [18] reported the use of a biodegradable polymeric matrix based on cassava starch, gelatin, polyvinyl alcohol, and glycerol as a plasticizer; they used the mixture as coatings for maize seeds and confirmed the ability of this material to sustain *A. brasilense* viability for up to 15 days after bacterial immobilization. This biodegradable coating was considered a promising low-cost, biodegradable, and renewable source material to be used in agriculture.

Perez et al. [22] reported the use of starch-chitosan beads for the release of *A. brasilense* and *P. fluorescens*. The biopolymeric beads loaded with the microorganisms were stored at room temperature, and they preserved the cells’ viability for a long period. After 1 year of storage, the recovery of bacteria from chitosan-starch beads was in the order of 10^9^ and 10^8^ CFU per gram for A. *brasilense* and *P. fluorescens*, respectively.

Saberi-Riseh et al. [103] reported that starch has some advantages compared to other biopolymers, including carboxymethyl cellulose, chitosan, alginate, gelatin, gums, and whey protein. Starch has a low cost, and it is more available than carboxymethyl cellulose and chitosan. Compared to alginate, the extraction process of starch from different sources is easier, and the high porosity and low mechanical strength of alginate beads can be a problem in the encapsulation of PGPB. Starch also has some advantages compared to gelatin, which requires higher concentrations to obtain a polymeric network, and the melting point of gelatin near room temperature (35 °C) can result in melting depending on storage conditions.

### 3.3. Polymeric Matrices Based on Chitosan

Chitosan is a renewable source of cationic and biodegradable polymer produced from the alkaline deacetylation of chitin [22]. Chitin can be obtained from several sources, including wastes of arthropod shells (shrimps, lobsters, crabs, crayfish) processed by the fishing industry, fungal cell walls, and plants [110]. It is constituted of two subunits, D-glucosamine and N-acetyl-D-glucosamine, which are bounded by β-(1→4) glycosidic linkages [117].

Ramírez et al. [118] reported that chitosan can protect plants from pests and diseases before and after a harvest and can enhance beneficial symbiotic plant-microorganism interactions, regulating plant growth and development. They also reported that the chelating nature of chitosan can be helpful for nutrient and mineral sequestration, making them available for uptake by plants.

Chitosan has been increasingly studied as a potential carrier for PGPB encapsulation, because it has many properties that are very interesting for use in agricultural systems, such as: (1) Inducing defense responses against phytopathogens by triggering the production of several defensive compounds. Chitosan also increases antimicrobial properties, promotes soil remediation, and activates defense mechanisms in plants growing in field conditions; (2) Promotion of plant growth; (3) Conditioning of the soil, by preventing the leaching of anionic nutrient fertilizers, such as phosphates and nitrates, also by stimulating the activity of beneficial microorganisms and enhancing the water retention properties of the soil [119]. Some authors reported the use of chitosan as a carrier for PGPB to obtain novel biofertilizers [120,121,122,123] or biopesticides [123,124], with very promising results.

Chanratana et al. [120] compared wet chitosan, dry chitosan, wet alginate, and dry alginate as carriers for *Methylobacterium oryzae*, which was employed for tomato growth promotion. The optimal immobilization condition was obtained for wet chitosan (1.5% solution) prepared at pH 5.5–6.0 and 90 min of contact time, resulting in a survivability of bacteria of 80% after 90 days of storage at 4 °C. Plants inoculated with this formulation had a 1.3-fold increase in shoot and root length and dry weight compared to other treatments. Chanratana et al. [121] reported that chitosan-immobilized *M. oryzae* increased tomato plant dry weight, nutrient uptake (N, P, K, and Mg^2+^), photosynthetic efficiency, and decreased electrolyte leakage under salt stress conditions.

The encapsulation of *B. licheniformis* in alginate-chitosan nanoparticle beads supplemented with rice starch did not affect the plant beneficial traits of the isolate, such as IAA production, nitrogen fixation, and ACC deaminase activity, enhancing the growth of chilli plants effectively compared to the free-living cells. In addition, the encapsulated *B. licheniformis* suppressed the disease caused by *Sclerotium rolfsii* in chilli plants [122].

*Streptomyces fulvissimus* was encapsulated in a chitosan-gellan gum matrix and resulted in a material with antimicrobial action against *Gaemannomyces graminis*, one of the most dangerous fungal diseases to wheat. The main mechanisms of *S. fulvissimus* include the production of antifungal antibiotics, the production of siderophores, and the production of chitinase and glucanase. Chitosan and gellan gum protected the bacteria and increased their survival rate in storage at room temperature [123].

### 3.4. Polymeric Matrices Based on Gelatin

Gelatin is a low-cost protein obtained through the acid or alkaline hydrolysis of collagen from the skin, bones, or connective tissue of animals, consisting of a large number of glycine, proline, and 4-hydroxyproline residues. Gelatin exhibits amphoteric behavior due to the presence of both basic and acidic groups [125]. It is easily soluble in water at an average temperature of 40 °C, forming a viscous solution by chain association and three-dimensional network formation, resulting in gels on cooling below 35 °C; above this temperature, gelatin exists as a single molecule because it is unable to form interchain hydrogen bonds [126,127,128,129]. Gelatin films are clear, flexible, strong, and oxygen permeable [129].

Gelatin, despite having a massive application in pharmaceutical and food industries, is still not exploited like other biopolymers for the encapsulation of PGPB. Some authors reported the use of gelatin in mixtures with alginate [98,130,131]. Using alginate in a mixture with gelatin to encapsulate *Mesorhizobium ciceri* and *Bradyrhizobium japonicum* increased the number of nodules formed in chickpea and soybean plants in comparison with non-inoculated plants [130]. *B. subtilis* was encapsulated in alginate-gelatin microspheres, and the viability of encapsulated PGPB could be preserved at more than 10^8^ CFU/mL after 120 days of storage at 25 °C [131].

## 4. Conclusions

The use of biopolymeric matrices as carriers for plant-growth-promoting bacteria in agricultural systems is a topic that is currently under great discussion by the scientific community. Encapsulation of these bacteria has been shown to be an effective method for protecting them from unsuitable environments. Many relevant results have been published, and alginate-based matrices are the most studied, but the use of starch from different sources is also very promising, considering its low cost and high availability. *A. brasilense* encapsulated in alginate matrices is the most studied formulation.

It is important to highlight that these biofertilizers and biopesticides play an important role in the establishment of sustainable and modern agriculture, which can support the increase in the world’s population and an associated increase in demand for food and energy in the next few decades with much lower environmental harm than conventional inputs. These new products are technological solutions for replacing deleterious chemical fertilizers and pesticides, maintaining soil fertility and stability, and improving crop productivity and food security.

Finally, in the near future, scale-up studies will have to provide new information about the large-scale production of these materials as well as their application in the field under different stress conditions.

## Figures and Tables

**Table 1:** Examples of PGPB mechanisms of action under different stresses and their improvement of plant growth.

Mechanism of Action	Microorganism	Plant	Stress Condition	Reference
Production of phytohormones ^1,2^ Production of antioxidants Production of siderophores	*Acinetobacter beijerinckii*	Soybean	Heavy metal (Cr)	[30]
Production of phytohormones ^1,2^ Production of antioxidants Production of siderophores	*Raoultella planticola*	Soybean	Heavy metal (Cr)	[30]
Biological nitrogen fixation	*Bradyrhizobium* spp	Soybean	-	[38,39]
Biological nitrogen fixation	*Azospirillum brasilense*	Maize	-	[40]
Production of phytohormones Improvement of water content	*A. brasilense*	Wheat	Drought	[41]
Biological nitrogen fixation Production of phytohormones ^2^	*A. brasilense*	Maize, wheat and rice	-	[42]
Biological nitrogen fixation	*Bacillus subtilis*	Maize and sorghum	-	[43]
Production of siderophores Production of antibiotics	*B. subtillis*	Pepper	Biotic	[44]
Biological nitrogen fixation Production of phytohormones ^2^ Production of exopolysaccharides Production of siderophores	*B. subtilis* *B. paralicheniformis* *Pseudomonas putida*	Maize	-	[45]
Production of phytohormones ^3^ Production of antioxidants	*P. putida*	Soybean	Salinity and drought	[46]
Production of exopolysaccharides	*P.putida*	Sunflower	Drought	[47]
Phosphate solubilization	*B. polymyxa*	Tomato	Drought	[48]
Phosphate solubilization ACC deaminase production	*B. thuringiensis*	Wheat	Drought	[49]
Production of antioxidants	*B. thuringiensis*	Lavender	Drought	[50]
ACC deaminase production	*Enterobacter mori* *E. asburiae* *E. ludwigii*	Wheat	Drought	[51]
Production of phytohormones ^3^	*Burkholdera cepacia* *Acinetobacter calcoaceticus*	Cucumber	Salinity	[52]
Production of phytohormones ^2^ Production of siderophores Phosphate solubilization	*Enterobacter sp*. *Pseudomonas* sp.	Tomato	-	[53]
Production of antibiotics	*B. amyloliquefaciens*	Wheat	Biotic *Fusarium graminearum*	[54]
Improvements in root architecture ACC deaminase production	*P. fluorescens*	Signalgrass	-	[55]
Phosphate solubilization Production of phytohormones ^2^ Production of siderophores Production of antibiotics Production of volatile organic compounds and ammonia	*P. aeruginosa*	Tomato, potato, taro, and strawberry	Biotic	[56]
Biological nitrogen fixation Phosphate solubilization Production of phytohormones ^2^ Production of chitinases	*B. velezensis*	Jujube fruits	Biotic	[57]
Production of antioxidants Production of siderophores	*B. simplex*	Pea	Heavy metal (Pb)	[58]
Phosphate solubilization Production of phytohormones ^2,3^ ACC deaminase production Production of exopolysaccharides	*Klebsiella* sp.	Mung bean	Salinity pH	[59]
Production of antioxidants	*Azotobacter chroococcum*	Maize	Drought	[60]

^1^ —Gibberellins; ^2^ —Abscisic acid; ^3^ —IAA.

## Data Availability

Not applicable.

## References

[1] FAO, IFAD, UNICEF, WFP, WHO (2022). The State of Food Security and Nutrition in the World 2022. Repurposing Food and Agricultural Policies to Make Healthy Diets More Affordable.

[2] Abbass K., Qasim M.Z., Song H., Murshed S., Mahmood H., Younis I. (2022). A Review of the Global Climate Change Impacts, Adaptation. Environ. Sci. Pollut. Res..

[3] Gupta A., Mishra R., Rai S., Bano A., Pathak N., Fujita M., Kumar M., Hasanuzzaman M. (2022). Mechanistic Insights of Plant Growth Promoting Bacteria Mediated Drought and Salt Stress Tolerance in Plants for Sustainable Agriculture. Int. J. Mol. Sci..

[4] Cao M., Narayanan M., Shi X., Chen X., Li Z., Ma Y. (2023). Optimistic Contributions of Plant Growth-Promoting Bacteria for Sustainable Agriculture and Climate Stress Alleviation. Environ. Res..

[5] Pörtner H.O., Roberts D.C., Tignor M., Poloczanska E.S., Mintenbeck K., Alegría A., Craig M., Langsdorf S., Löschke S., Möller V. (2022). Climate Change 2022: Impacts, Adaptation and Vulnerability. Contribution of Working Group ii to the Sixth Assessment Report of the Intergovernmental Panel on Climate Change.

[6] Sohail M.T., Mustafa S., Ali M.M., Riaz S. (2022). Agricultural Communities’ Risk Assessment and the Effects of Climate Change: A Pathway toward Green Productivity and Sustainable Development. Front. Environ. Sci..

[7] Muhie S.H. (2022). Novel Approaches and Practices to Sustainable Agriculture. J. Agric. Food Res..

[8] Cernev T., Fenner R. (2020). The Importance of Achieving Foundational Sustainable Development Goals in Reducing Global Risk. Futures.

[9] Jaiswar A., Varshney D., Kaushik V., Sharma N., Bedi A., Bhat R.A., Butnariu M., Dar G.H., Hakeem K.R. (2023). Plant-Associated Bacteria in Ecosystems Functioning and Sustainability. Microbial Bioremediation.

[10] Gamalero E., Bona E., Glick B.R. (2022). Current Techniques to Study Beneficial Plant-Microbe Interactions. Microorganisms.

[11] Olanrewaju O.S., Glick B.R., Babalola O.O. (2017). Mechanisms of Action of Plant Growth Promoting Bacteria. World J. Microbiol. Biotechnol..

[12] Pirzada T., Farias B.V., Mathew R., Guenther R.H., Byrd M.V., Sit T.L., Khan S.A. (2020). Recent Advances in Biodegradable Matrices for Active Ingredient Release in Crop Protection: Towards Attaining Sustainability in Agriculture. COCIS.

[13] Goswami D., Thakker J.N., Dhandhukia P.C. (2016). Portraying Mechanics of Plant Growth Promoting Rhizobacteria (PGPR): A Review. Cogent Food Agric..

[14] Saberi-Riseh R., Ebrahimi-Zarandi M., Gholizadeh Vazvani M., Skorik Y.A. (2021). Reducing drought stress in plants by Encapsulating Plant Growth-Promoting Bacteria with Polysaccharides. Int. J. Mol. Sci..

[15] Elnahal A.S.M., Saadoney M.T., Saad A.M., Desoky E.S.M., Tahan A.M., Rady M.M., Abuqamar S., Tarabily K.A. (2022). The Use of Microbial Inoculants for Biological Control, Plant Growth Promotion, and Sustainable Agriculture: A Review. Eur. J. Plant. Pathol..

[16] Balla A., Silini A., Cherif-Silini H., Chenari Bouket A., Alenezi F.N., Belbahri L. (2022). Recent Advances in Encapsulation Techniques of Plant Growth-Promoting Microorganisms and their Prospects in the Sustainable Agriculture. Appl. Sci..

[17] Marcelino P.R.F., Milani K.M., Mali S., Santos O.J.A.P., de Oliveira A.L.M. (2016). Formulations of Polymeric Biodegradable Low-Cost Foam by Melt Extrusion to Deliver Plant Growth-Promoting Bacteria in Agricultural Systems. Appl. Microbiol. Biotechnol..

[18] Vercelheze A.E.S., Marim B.M., Oliveira A.L.M., Mali S. (2019). Development of Biodegradable Coatings for Maize Seeds and their Application for *Azospirillum brasilense* Immobilization. Appl. Microbiol. Biotechnol..

[19] Juric S., Ðermic E., Topolovec-Pintaric S., Bedek M., Vincekovic M. (2019). Physicochemical Properties and Release Characteristics of Calcium Alginate Microspheres Loaded with *Trichoderma viride* spores. J. Integr. Agric..

[20] Bashan Y., Hernandez J.P., Leyva L.A., Bacilio M. (2002). Alginate Microbeads as Inoculant Carriers for Plant Growth-Promoting Bacteria. Biol. Fertil. Soils.

[21] Young C.C., Rekha P.D., Lai W.A., Arun A.B. (2006). Encapsulation of Plant Growth-Promoting Bacteria in Alginate Beads Enriched with Humic Acid. Biotechnol. Bioeng..

[22] Perez J.J., Francois N.J., Maroniche G.A., Borrajo M.P., Pereyra M.A., Creus C.M. (2018). A Novel, Green, Low-Cost Chitosan-Starch Hydrogel as Potential Delivery System for Plant Growth-Promoting Bacteria. Carbohydr. Polym..

[23] Lobo C.B., Tomás M.S.J., Viruel E., Ferrero M.A., Lucca M.E. (2018). Development of Low-Cost Formulations of Plant Growth-Promoting Bacteria to Be Used as Inoculants in Beneficial Agricultural Technologies. Microbiol. Res..

[24] Fadiji A.E., Santoyo G., Yadav A.N., Babalola O.O. (2022). Efforts Towards Overcoming Drought Stress in Crops: Revisiting the Mechanisms Employed by Plant Growth-Promoting Bacteria. Front. Microbiol..

[25] Sati D., Pande V., Pandey S.C., Samant M. (2022). Recent Advances in PGPR and Molecular Mechanisms Involved in Drought Stress Resistance. J. Soil Sci. Plant Nutr..

[26] Abdelaal K., AlKahtani M., Attia K., Hafez Y., Király L., Künstler A. (2021). The Role of Plant Growth-Promoting Bacteria in Alleviating the Adverse Effects of Drought on Plants. Biology.

[27] Ortiz-Castro R., Campos-García J., López-Bucio J. (2020). *Pseudomonas putida* and *Pseudomonas fluorescens* influence *Arabidopsis* Root System Architecture through an Auxin Response Mediated by Bioactive Cyclodipeptides. J. Plant Growth Regul..

[28] Glick B.R. (1995). The Enhancement of Plant Growth by Free-Living Bacteria. Can. J. Microbiol..

[29] Stegelmeier A.A., Rose D.M., Joris B.R., Glick B.R. (2022). The Use of PGPB to Promote Plant Hydroponic Growth. Plants.

[30] Hussain A., Shah M., Hamayun M., Iqbal A., Qadir M., Alataway A., Dewidar A.Z., Elansary H.O., Lee I. (2023). Phytohormones Producing Rhizobacteria Alleviate Heavy Metals Stress in Soybean through Multilayered Response. Microbiol. Res..

[31] Mishra P., Mishra J., Arora N.K. (2021). Plant Growth Promoting Bacteria for Combating Salinity Stress in Plants—Recent Developments and Prospects: A Review. Microbiol. Res..

[32] Ngalimat M.S., Mohd Hata E., Zulperi D., Ismail S.I., Ismail M.R., Mohd Zainudin N.A.I., Saidi N.B., Yusof M.T. (2021). Plant Growth-Promoting Bacteria as an Emerging Tool to Manage Bacterial Rice Pathogens. Microorganisms.

[33] Orozco-Mosqueda M.D.C., Flores A., Rojas-Sánchez B., Urtis-Flores C.A., Morales-Cedeño L.R., Valencia-Marin M.F., Chávez-Avila S., Rojas-Solis D., Santoyo G. (2021). Plant Growth-Promoting Bacteria as Bioinoculants: Attributes and Challenge for Sustainable Crop Improvement. Agronomy.

[34] Ali B., Hafeez A., Javed M.A., Afridi M.S., Abbasi H.A., Qayyum A., Batool T., Ullah A., Marc R.A., Jaouni S.K. (2022). Role of Endophytic Bacteria in Salinity Stress Amelioration by Physiological and Molecular Mechanisms of Defense: A Comprehensive Review. S. Afri. J. Bot..

[35] Morales C.L.R., Mosqueda O.M.C., Lara L.P.D., Cota P.F.I., Villalobos S.S., Santoyo G. (2020). Plant Growth-Promoting Bacterial Endophytes as Biocontrol Agents of Pre- and Post-Harvest Diseases: Fundamentals, Methods of Application and Future Perspectives. Microbiol. Res..

[36] Bashan Y., Bashan L.E., Prabhu S.R., Hernandez J.P. (2014). Advances in Plant Growth-Promoting Bacterial Inoculant Technology: Formulations and Practical Perspectives (1998–2013). Plant Soil.

[37] Santos M.S., Rodrigues T.F., Nogueira M.A., Hungria M. (2021). The Challenge of Combining High Yields with Environmentally Friendly Bioproducts: A Review on the Compatibility of Pesticides with Microbial Inoculants. Agronomy.

[38] Hungria M., Mendes I.C. (2015). Nitrogen Fixation with Soybean: The Perfect Symbiosis?. Biological Nitrogen Fixation.

[39] Freitas V.F., Cerezini P., Hungria M., Nogueira M.A. (2022). Strategies to Deal with Drought-Stress in Biological Nitrogen Fixation in Soybean. Appl. Soil Ecol..

[40] Martins M.R., Jantalia C.P., Reis V.M. (2018). Impact of Plant Growth-Promoting Bacteria on Grain Yield, Protein Content, and Urea-15 N Recovery by Maize in a Cerrado Oxisol. Plant Soil.

[41] Furlan F., Saatkamp K., Volpiano C.G., De Assis Franco F., Dos Santos M.F., Vendruscolo E.C.G. (2017). Plant Growth-Promoting Bacteria Effect in Withstanding Drought in Wheat Cultivars. Sci. Agrar..

[42] Fukami J., Cerezini P., Hungria M. (2018). *Azospirillum*: Benefits that go far beyond biological nitrogen fixation. AMB Express.

[43] Aquino J.P.A., Macedo F.B.J., Antunes J.E.L., Figueiredo M.V.B., Neto F.A., Araujo A.S.F. (2019). Plant Growth-Promoting Endophytic Bacteria on Maize and Sorghum|1. Pesqui. Agropecu. Trop..

[44] Lee K.J., Kamala-Kannan S., Sub H.S., Seong C.K., Lee G.W. (2008). Biological Control of *Phytophthora* Blight in Red Pepper (*Capsicum annuum* L.) using *Bacillus subtilis*. World J. Microbiol. Biotechnol..

[45] Abadi V.A.J.M., Sepehri M., Rahmani H.A., Zarei M., Ronaghi A., Taghavi S.M., Shamshiripour M. (2020). Role of Dominant Phyllosphere Bacteria with Plant Growth–Promoting Characteristics on Growth and Nutrition of Maize (*Zea mays* L.). J. Soil Sci. Plant Nutr..

[46] Kang S.M., Radhakrishnan R., Khan A.L., Kim M.J., Park J.M., Kim B.R., Shin D.H., Lee I.J. (2014). Gibberellin Secreting Rhizobacterium, *Pseudomonas putida* H-2-3 Modulates the Hormonal and Stress Physiology of Soybean to Improve the Plant Growth under Saline and Drought Conditions. Plant Physiol. Biochem..

[47] Sandhya V.Z.A.S., SK Z.A., Grover M., Reddy G., Venkateswarlu B. (2009). Alleviation of Drought Stress Effects in Sunflower Seedlings by the Exopolysaccharides Producing *Pseudomonas putida* strain GAP-p45. Biol. Fertil. Soils.

[48] Shintu P.V., Jayaram K.M. (2015). Phosphate Solubilising Bacteria (*Bacillus polymyxa*)—An Effective Approach to Mitigate Drought in Tomato (*Lycopersicon esculentum* Mill.). Trop. Plant Res..

[49] Timmusk S., El-Daim I.A.A., Copolovici L., Tanilas T., Kännaste A., Behers L., Nevo E., Seisenbaeva G., Stenström E., Niinemets Ü. (2014). Drought-Tolerance of Wheat Improved by Rhizosphere Bacteria from Harsh Environments: Enhanced Biomass Production and Reduced Emissions of Stress Volatiles. PLoS ONE.

[50] Armada E., Probanza A., Roldán A., Azcón R. (2016). Native Plant Growth Promoting Bacteria *Bacillus thuringiensis* and Mixed or Individual Mycorrhizal Species Improved Drought Tolerance and Oxidative Metabolism in *Lavandula dentata* Plants. J. Plant Physiol..

[51] Zhang G., Sun Y., Sheng H., Li H., Liu X. (2018). Effects of the Inoculations Using Bacteria Producing ACC Deaminase on Ethylene Metabolism and Growth of Wheat Grown under Different Soil Water Contents. Plant Physiol. Biochem..

[52] Kang S.M., Khan A.L., Waqas M., You Y.H., Kim J.H.J.G., Kim J.H.J.G., Hamayun M., Lee I.J. (2014). Plant Growth-Promoting Rhizobacteria Reduce Adverse Effects of Salinity and Osmotic Stress by Regulating Phytohormones and Antioxidants in *Cucumis sativus*. J. Plant Interact..

[53] Zuluaga M.Y.A., Milani K.M.L., Miras-Moreno B., Lucini L., Valentinuzzi F., Mimmo T., Pii Y., Cesco S., Rodrigues E.P., de Oliveira A.L.M. (2020). Inoculation with Plant Growth-Promoting Bacteria Alters the Rhizosphere Functioning of Tomato Plants. Appl. Soil Ecol..

[54] Gu Q., Yang Y., Yuan Q., Shi G., Wu L., Lou Z., Huo R., Wu H., Borriss R., Gao X. (2017). Bacillomycin D Produced by *Bacillus amyloliquefaciens* is Involved in the Antagonistic Interaction with the Plant-Pathogenic Fungus *Fusarium graminearum*. Appl. Environ. Microbiol..

[55] Hungria M., Rondina A.B.L., Nunes A.L.P., Araújo R.S., Nogueira M.A. (2021). Seed and Leaf-Spray Inoculation of PGPR in Brachiarias (*Urochloa* spp.) as an Economic and Environmental Opportunity to Improve Plant Growth, Forage Yield and Nutrient Status. Plant Soil.

[56] Ghadamgahi F., Tarighi S., Taheri P., Saripella G.V., Anzalone A., Kalyandurg P.B., Catara V., Ortiz R., Vetukuri R.R. (2022). Plant Growth-Promoting Activity of *Pseudomonas Aeruginosa* FG106 and its Ability to Act as a Biocontrol Agent against Potato, Tomato and Taro Pathogens. Biology.

[57] Choi S.I., Ajuna H.B., Won S.J., Choub V., Kim C.W., Moon J.H., Ahn Y.S. (2023). The Insecticidal Potential of *Bacillus velezensis* CE 100 against *Dasineura jujubifolia* Jiao & Bu (Diptera: Cecidomyiidae) Larvae Infestation and its Role in the Enhancement of Yield and Fruit Quality of Jujube (*Zizyphus jujuba* Miller var. inermis Rehder). Crop Protec..

[58] Chamekh A., Kharbech O., Fersi C., Liman R.D., Brandt K.K., Djebali W., Chouari R. (2023). Insights on Strain 115 Plant Growth-Promoting Bacteria Traits and its Contribution in Lead Stress Alleviation in Pea (*Pisum sativum* L.) Plants. Arch. Microbiol..

[59] Gohil R.B., Raval V.H., Panchal R.R., Rajput K.N. (2023). Plant Growth Promoting Activities and Effect of Fermented Panchagavya Isolate *Klebsiella* sp. PG-64 on *Vigna radiata*. World J. Microbiol. Biotechnol..

[60] Tyagia J., Mishra A., Kumari S., Singh S., Agarwal H., Pudake R.M., Varma A., Joshi N.C. (2023). Deploying a Microbial Consortium of *Serendipita indica*, *Rhizophagus intraradices*, and *Azotobacter chroococcum* to Boost Drought Tolerance in Maize. Environ. Exp. Bot..

[61] Santos M.S., Nogueira M.A., Hungria M. (2021). Outstanding Impact of *Azospirillum brasilense* Strains Ab-V5 and Ab-V6 on the Brazilian Agriculture: Lessons that Farmers are Receptive to Adopt New Microbial Inoculants. Rev. Bras. Ciênc. Solo.

[62] Farquharson E.A., Ballard R.A., Herridge D.F., Ryder M.H., Denton M.D., Webster A., Yates R.J., Seymour N.P., Deaker R.J., Hartley E. (2022). Inoculating Legumes: Practice and Science.

[63] MAPA—Ministério da Agricultura, Pecuária e Abastecimento (2004). Instrução Normativa No 5, de 6 de Agosto de 2004.

[64] MAPA—Ministério da Agricultura, Pecuária e Abastecimento (2011). Instrução Normativa No 13, de 24 de Março de 2011.

[65] Hungria M., Campo R.J., Izaguirre-Mayoral M.L., Labandera C., Sanjuan J. (2007). Inoculantes Microbianos: Situação no Brasil. Biofertilizantes en Iberoamérica: Visión Técnica, Científica y Empresarial.

[66] Fusco G.M., Nicastro R., Rouphael Y., Carillo P. (2022). The effects of the microbial biostimulants approved by EU regulation 2019/1009 on yield and quality of vegetable crops. Foods.

[67] EC (2019). REGULATION (EU) 2019/1009 of the European Parliament and of the Council of 5 June 2019 Laying down Rules on the Making Available on the Market of EU Fertilising Products and Amending Regulations (EC) No 1069/2009 and (EC) No 1107/2009 and Repealing Regulation (EC) No 2003/2003. https://eur-lex.europa.eu/legal-content/EN/TXT/?uri=celex%3A32019R1009.

[68] Schoebitz M., López M.D., Roldán A. (2013). Bioencapsulation of Microbial Inoculants for Better Soil–Plant Fertilization. A Review. Agron. Sustain. Dev..

[69] Malik R., Saxena R., Warkar S.G. (2022). Biopolymer-Based Biomatrices—Organic Strategies to Combat Micronutrient Deficit for Dynamic Agronomy. ChemistrySelect.

[70] John R.P., Tyagi R.D., Brar S.K., Surampalli R.Y., Prévost D. (2011). Bio-Encapsulation of Microbial Cells for Targeted Agricultural Delivery. Crit. Rev. Biotechnol..

[71] Vemmer M., Patel A.V. (2013). Review of Encapsulation Methods Suitable for Microbial Biological Control Agents. Biol. Control..

[72] Chaudhary T., Dixit M., Gera R., Shukla A.K., Prakash A., Gupta G., Shukla P. (2020). Techniques for Improving Formulations of Bioinoculants. 3Biotech.

[73] Szekalska M., Puciłowska A., Szymańska E., Ciosek P., Winnicka K. (2016). Alginate: Current Use and Future Perspectives in Pharmaceutical and Biomedical Applications. Int. J. Polym. Sci..

[74] Karmakar S., Manna S., Kabiraj S., Jana S. (2022). Recent progress in alginate-based carriers for ocular targeting of therapeutics. FHFH.

[75] Pereira L., Cotas J., Pereira L. (2020). Introductory Chapter: Alginates—A General Overview. Alginates—Recent Uses of This Natural Polymer.

[76] Martínez-Cano B., Mendoza-Meneses C.J., García-Trejo J.F., Macías-Bobadilla G., Aguirre-Becerra H., Soto-Zarazúa G.M., Feregrino-Pérez A.A. (2022). Review and Perspectives of the Use of Alginate as a Polymer Matrix for Microorganisms Applied in Agro-Industry. Molecules.

[77] Bashan Y. (1986). Alginate Beads as Synthetic Inoculant Carriers for Slow Release of Bacteria that Affect Plant Growth. Appl. Environ. Microbiol..

[78] Bashan Y., Gonzalez L.E. (1999). Long-Term Survival of the Plant-Growth-Promoting Bacteria *Azospirillum brasilense* and *Pseudomonas fluorescens* in Dry Alginate Inoculant. Appl. Microbiol. Biotechnol..

[79] Russo A., Basaglia M., Tola E., Casella S. (2001). Survival, Root Colonization and Biocontrol Capacities of *Pseudomonas fluorescens* F113 LacZY in Dry Alginate Microbeads. J. Ind. Microbiol. Biotechnol..

[80] Joe M.M., Saravanan V.S.V., Islam M.R., Sa T. (2014). Development of Alginate-Based Aggregate Inoculants of *Methylobacterium* sp. and *Azospirillum brasilense* Tested Under in vitro Conditions to Promote Plant Growth. J. Appl. Microbiol..

[81] El-Komy H. (2005). Coimmobilization of *Azospirillum lipoferum* and *Bacillus megaterium* for Successful Phosphorus and Nitrogen Nutrition of Wheat Plants. Food Technol. Biotechnol..

[82] Yabur R., Bashan Y., Hernández-Carmona G. (2007). Alginate from the Macroalgae *Sargassum sinicola* as a Novel Source for Microbial Immobilization Material in Wastewater Treatment and Plant Growth Promotion. J. Appl. Phycol..

[83] Trivedi P., Pandey A. (2008). Recovery of Plant Growth-Promoting Rhizobacteria from Sodium Alginate Beads after 3 Years following Storage at 4 °C. J. Ind. Microbiol. Biotechnol..

[84] Wu Z., Zhao Y., Kaleem I., Li C. (2011). Preparation of Calcium–Alginate Microcapsuled Microbial Fertilizer Coating *Klebsiella oxytoca* Rs-5 and its Performance under Salinity Stress. Eur. J. Soil Biol..

[85] Schoebitz M., Simonin H., Poncelet D. (2012). Starch Filler and Osmoprotectants Improve the Survival of Rhizobacteria in Dried Alginate Beads. J. Microencapsul..

[86] Reetha D., Kumaresan G., John Milton D. (2014). Studies to Improve the Shelf Life of *Azospirillum lipoferum* Immobilized in Alginate Beads. Int. J. Recent Sci. Res..

[87] Wang H.Q., Hua F., Zhao Y.C., Li Y., Wang X. (2014). Immobilization of *Pseudomonas* sp. DG17 onto Sodium Alginate–Attapulgite–Calcium Carbonate. Biotechnol. Biotechnol. Equip..

[88] Farhat M.B., Taktek S., Chouayekh H. (2014). Encapsulation in Alginate Enhanced the Plant Growth Promoting Activities of Two Phosphate Solubilizing Bacteria Isolated from the Phosphate Mine of Gafsa. Net J. Agric. Sci..

[89] Forestier S., Alvarado G., Badjel Badjel S., Lesueur D. (2001). Effect of *Rhizobium* Inoculation Methodologies on Nodulation and Growth of *Leucaena leucocephala*. World J. Microbiol. Biotechnol..

[90] Gurley H.G., Zdor R.E. (2005). Differential Rhizosphere Establishment and Cyanide Production by Alginate-Formulated Weed-Deleterious Rhizobacteria. Curr. Microbiol..

[91] Saberi-Riseh R., Moradi-Pour M. (2020). The Effect of *Bacillus subtilis* Vru1 Encapsulated in Alginate–Bentonite Coating Enriched with Titanium Nanoparticles against *Rhizoctonia solani* on Bean. Int. J. Biol. Macromol..

[92] Panichikkal J., Prathap G., Nair R.A., Krishnankutty R.E. (2021). Evaluation of Plant Probiotic Performance of *Pseudomonas* sp. Encapsulated in Alginate Supplemented with Salicylic Acid and Zinc Oxide Nanoparticles. Int. J. Biol. Macromol..

[93] Souza-Alonso P., Rocha M., Rocha I., Ma Y., Freitas H., Oliveira R.S. (2021). Encapsulation of *Pseudomonas libanensis* in Alginate Beads to Sustain Bacterial Viability and Inoculation of *Vigna unguiculata* under Drought Stress. Biotech.

[94] Zhang W., Zheng L., Lang D., Zhang X., Ma X., Li X., Zhang X. (2023). Eco-friendly Bio-Encapsulation from Sodium Alginate-Trehalose-Kaolin and its Performance Evaluation in Improving Plant Growth under Salt or/and Drought Conditions. Int. J. Biol. Macromol..

[95] Hernandéz-Montiel L.G., Contreras C.J.C., Amador B.M., Hernández L.V., Aguilar E.E.Q. (2017). Contreras, R.G.C. Efficiency of Two Inoculation Methods of *Pseudomonas putida* on Growth and Yield of Tomato Plants. J. Soil Sci. Plant Nutr..

[96] Zago S.L., dos Santos M.F., Konrad D., Fiorini A., Rosado F.R., Missio R.F., Vendruscolo E.C.G. (2019). Shelf Life of *Azospirillum brasilense* in Alginate Beads Enriched with Trehalose and Humic Acid. J. Agric. Sci..

[97] Wiwattanapatapee R., Chumthong A., Pengnoo A., Kanjanamaneesathian M. (2013). Preparation and Evaluation of *Bacillus megaterium*-Alginate Microcapsules for Control of Rice Sheath Blight Disease. World J. Microbiol. Biotechnol..

[98] Pour M.M., Riseh R.S., Mohammadinejad R., Hosseini A. (2019). Investigating the Formulation of Alginate-Gelatin Encapsulated *Pseudomonas fluorescens* (VUPF5 and T17–4 Strains) for Controlling *Fusarium solani* on Potato. Int. J. Biol. Macromol..

[99] Jung G., Mugnier J., Diem H.G. (1982). Polymer-Entrapped Rhizobium as an Inoculant for Legumes. Plant Soil.

[100] Fages J. (1992). An industrial view of *Azospirillum* inoculants: Formulation and Application Technology. Symbiosis.

[101] Szopa D., Mielczarek M., Skrzypczak D., Izydorczyk G., Mikula K., Chojnacka K., Krowiak A.W. (2022). Encapsulation Efficiency and Survival of Plant Growth-Promoting Microorganisms in an Alginate-Based Matrix—A Systematic Review and Protocol for a Practical Approach. Ind. Crops Prod..

[102] Rohman S., Kaewtatip K., Kantachote D., Tantirungkij M. (2021). Encapsulation of *Rhodopseudomonas palustris* KTSSR54 Using Beads from Alginate/Starch Blends. J. Appl. Polym. Sci..

[103] Saberi-Riseh R., Hassanisaadi M., Vatankhaha M., Kennedy J.F. (2023). Encapsulating Biocontrol Bacteria with Starch as a Safe and Edible Biopolymer to Alleviate Plant Diseases: A Review. Carbohydr. Polym..

[104] Vejan P., Khadiran T., Abdullah R., Ismail S., Dadrasnia A. (2019). Encapsulation of Plant Growth Promoting Rhizobacteria—Prospects and Potential in Agricultural Sector: A Review. J. Plant Nutr..

[105] Falcão L.d.S., Coelho D.B., Veggi P.C., Campelo P.H., Albuquerque P.M., de Moraes M.A. (2022). Starch as a Matrix for Incorporation and Release of Bioactive Compounds: Fundamentals and Applications. Polymers.

[106] Tester R.F., Karkalas J., Qi X. (2004). Starch—Composition, Fine Structure and Architecture. J. Cereal Sci..

[107] Minakawa A.F.K., Faria-Tischer P.C.S., Mali S. (2019). Simple Ultrasound Method to Obtain Starch Micro- and Nanoparticles from Cassava, Corn and Yam Starches. Food Chem..

[108] Mali S., Grossmann M.V.S., Yamashita F. (2010). Filmes de Amido: Produção, Propriedades e Potencial de Utilização. Semin. Cienc. Agrar..

[109] Sueiro A.C., Faria-Tischer P.C.S., Lonni A.A.S.G., Mali S. (2016). Filmes Biodegradáveis de Amido de Mandioca, Pululana e Celulose Bacteriana. Quim. Nova.

[110] Mali S., Sakanaka L.S., Yamashita F., Grossmann M.V.E. (2005). Water Sorption and Mechanical Properties of Cassava Starch Films and their Relation to Plasticizing Effect. Carbohydr. Polym..

[111] Luo K., Adra H.J., Kim Y.-R. (2020). Preparation of Starch-Based Drug Delivery System through the Self-Assembly of Short Chain Glucans and Control of its Release Property. Carbohydr. Polym..

[112] Lu X., Li C., Huang Q. (2019). Combining in vitro Digestion Model with Cell Culture Model: Assessment of Encapsulation and Delivery of Curcumin in Milled Starch Particle Stabilized Pickering Emulsions. Int. J. Biol. Macromol..

[113] Cheng M., Yan X., Cui Y., Han M., Wang Y., Wang J., Zhang R., Wang X. (2022). Characterization and Release Kinetics Study of Active Packaging Films Based on Modified Starch and Red Cabbage Anthocyanin Extract. Polymers.

[114] Rodrigues G.D.M., Filgueiras C.T., Garcia V.A.D.S., De Carvalho R.A., Velasco J.I., Fakhouri F.M. (2020). Antimicrobial Activity and GC-MS Profile of Copaiba Oil for Incorporation into *Xanthosoma Mafaffa* Schott Starch-Based Films. Polymers.

[115] Tomsone L., Galoburda R., Kruma Z., Durrieu V., Cinkmanis I. (2020). Microencapsulation of Horseradish (*Armoracia rusticana* L.) Juice Using Spray-Drying. Foods.

[116] Ravichai K., Muangrat R. (2019). Effect of Different Coating Materials on Freeze-Drying Encapsulation of Bioactive Compounds from Fermented Tea Leaf Wastewater. J. Food Process. Preserv..

[117] Ferreira D.C.M., Souza A.L., Silveira J.V.W., Marim B.M., Giraldo G.A.G., Mantovan J., Mali S., Pelissari F.M. (2020). Chitosan Nanocomposites for Food Packaging Applications. Multifunctional Hybrid Nanomaterials for Sustainable Agri-Food and Ecosystems.

[118] Ramírez M.Á., Rodriguez A.T., Alfonso L., Peniche C. (2010). Chitin and its Derivatives as Biopolymers with Potential Agricultural Applications. Biotecnol. Aplic..

[119] Saberi-Riseh R., Tamanadar E., Hajabdollahi N., Vatankhah M., Thakur V.K., Skorik Y.A. (2022). Chitosan Microencapsulation of Rhizobacteria for Biological Control of Plant Pests and Diseases: Recent Advances and Applications. Rhizosphere.

[120] Chanratana M., Han G.H., Joe M.M., Choudhury A.R., Sundaram S., Halim M.A., Sa T. (2018). Evaluation of Chitosan and Alginate Immobilized *Methylobacterium oryzae* CBMB20 on Tomato Plant Growth. Arch. Agro. Soil Sci..

[121] Chanratana M., Joe M.M., Roy Choudhury A., Anandham R., Krishnamoorthy R., Kim K., Jeon S., Choi J., Choi J., Sa T.M. (2019). Physiological Response of Tomato Plant to Chitosan-Immobilized Aggregated *Methylobacterium oryzae* CBMB20 Inoculation under Salinity Stress. 3Biotech.

[122] Panichikkal J., Puthiyattil N., Raveendran A., Nair R.A., Krishnankutty R.E. (2021). Application of Encapsulated *Bacillus licheniformis* Supplemented with Chitosan Nanoparticles and Rice Starch for the Control of *Sclerotium rolfsii* in *Capsicum annuum* (L.) Seedlings. Curr. Microbiol..

[123] Saberi-Riseh R., Moradi-Pour M. (2021). A Novel Encapsulation of *Streptomyces fulvissimus* Uts22 by Spray Drying and its Biocontrol Efficiency against *Gaeumannomyces graminis*, the Causal Agent of Take-All Disease in Wheat. Pest Manag. Sci..

[124] Urena-Saborío H., Madrigal-Carballo S., Sandoval J., Vega-Baudrit J.R., Rodríguez-Morales A. (2017). Encapsulation of Bacterial Metabolic Infiltrates Isolated from Different *Bacillus* Strains in Chitosan Nanoparticles as Potential Green Chemistry based Biocontrol Agents against *Radopholus similis*. J. Renew. Mater..

[125] Rodríguez-Rodríguez R., Andrews E.H., Martínez C.V., Carvajal Z.Y.G. (2020). Composite Hydrogels Based on Gelatin, Chitosan and Polyvinyl Alcohol to Biomedical Applications: A Review. Int. J. Polym. Mater. Polym. Biomater..

[126] Laffleur F., Strasdat B. (2019). Gelatin-based formulations for dermal application. Eur. Polym. J..

[127] Wang C.S., Natale G., Virgilio N., Heuzey M.C. (2016). Synergistic Gelation of Gelatin B with Xanthan Gum. Food Hidrocoll..

[128] Rojas-Sánchez B., Guzmán-Guzmán P., Morales-Cedeño L.R., Orozco-Mosqueda M.d.C., Saucedo-Martínez B.C., Sánchez-Yáñez J.M., Fadiji A.E., Babalola O.O., Glick B.R., Santoyo G. (2022). Bioencapsulation of Microbial Inoculants: Mechanisms, Formulation Types and Application Techniques. Appl. Biosci..

[129] Pereira J.F., Lonni A.A.S.G., Mali S. (2021). Development of Biopolymeric Films with Addition of Vitamin C and Catuaba Extract as Natural Antioxidants. Prep. Biochem. Biotechnol..

[130] Shcherbakova E.N., Shcherbakov A.V., Rots P.Y., Gonchar L.N., Mulina S.A., Yahina L.M., Lactionov Y.V., Chebotar V.K. (2018). Inoculation Technology for Legumes Based on Alginate Encapsulation. Agron. Res..

[131] Tu L., He Y., Yang H., Wu Z., Yi L. (2015). Preparation and Characterization of Alginate-Gelatin Microencapsulated *Bacillus subtilis* SL-13 by Emulsification/Internal Gelation. J. Biomater. Sci. Polym..

